# Metabolite Changes in Orange Dead Leaf Butterfly *Kallima inachus* during Ontogeny and Diapause

**DOI:** 10.3390/metabo12090804

**Published:** 2022-08-27

**Authors:** Ming-Jie Li, Guo-Fang Jiang, Wei Wang

**Affiliations:** 1 College of Oceanology and Food Sciences, Donghai Campus, Quanzhou Normal University, Quanzhou 362000, China; 2 Wildlife Resource Conservation and Utilization Center, Guilinyang Campus, Qiongtai Normal University, Haikou 571100, China

**Keywords:** orange dead leaf butterfly, metabolism, metabolite changes, ontogeny, diapause, biomarkers

## Abstract

Holometabolism is a form of insect development which includes four life stages: egg, larva, pupa, and imago (or adult). The developmental change of whole body in metabolite levels of holometabolous insects are usually ignored and lack study. Diapause is an alternative life-history strategy that can occur during the egg, larval, pupal, and adult stages in holometabolous insects. *Kallima inachus* (Lepidoptera: Nymphalidae) is a holometabolous and adult diapausing butterfly. This study was intended to analyze metabolic changes in *K. inachus* during ontogeny and diapause through a non-targeted UPLC-MS/MS (ultra-performance liquid chromatograph coupled with tandem mass spectrometry) based metabolomics analysis. A variety of glycerophospholipids (11), amino acid and its derivatives (16), and fatty acyls (nine) are crucial to the stage development of *K. inachus*. 2-Keto-6-acetamidocaproate, N-phenylacetylglycine, Cinnabarinic acid, 2-(Formylamino) benzoic acid, L-histidine, L-glutamate, and L-glutamine play a potentially important role in transition of successive stages (larva to pupa and pupa to adult). We observed adjustments associated with active metabolism, including an accumulation of glycerophospholipids and carbohydrates and a degradation of lipids, as well as amino acid and its derivatives shifts, suggesting significantly changed in energy utilization and management when entering into adult diapause. Alpha-linolenic acid metabolism and ferroptosis were first found to be associated with diapause in adults through pathway analyses. Our study lays the foundation for a systematic study of the developmental mechanism of holometabolous insects and metabolic basis of adult diapause in butterflies.

## 1. Introduction

Holometabolism is a form of insect development. It means insects will go through several developmental stages (immature and mature stages) to adapted for different activities during ontogeny [1,2]. The majority of described insects are holometabolous [3]. Compare with paurometabolous insects, immature stages (i.e., larvae and pupae) of holometabolous insects are very different from the mature stages (i.e., adults and diapausing individuals) on morphology, behavior, biology, and biochemistry [4]. Metabolomics is an important analytical tool of systems biology, which can identify all detectable metabolites in a biological system [5]. It is used to reveal endogenous metabolite changes that are caused by environment stress, disease process, or gene function [6]. Several metabolomes have been generated to study the developmental changes for the hemolymph of lepidopteron. In tobacco hornworm (*Manduca sexta*), ^1^H-NMR result showed that the levels of many small molecule metabolites such as alanine, succinate, and fatty acid dramatically change in hemolymph during ontogeny [7]. Several studies suggest that the silkworm (*Bombyx mori*) exhibits specific metabolic pattern during ontogeny [8,9]. Lack of whole-body untargeted metabolome has existed as a problem for many years due to the limitations of ^1^H-NMR, and therefore hindered the in-depth studies in holometabolism. Butterflies are the one of the most widespread and widely recognizable insects in the world [10]. Butterflies are holometabolous and suitable for studying complete metamorphosis [11]. Unfortunately, the developmental changes in metabolite levels of butterfly during ontogeny are lack until now.

Diapause is a hormonally controlled state of metabolic suppression and cessation of development, allowing insects to survive periods of adverse conditions [12]. Studies over the past three decades have well defined the regulation of insect diapause on hormonal levels [13,14]. Currently, the research centers on metabolic adaptive mechanism underlying diapause at immature stage [15,16]. Reduced TCA (tricarboxylic acid) cycle activity was reported be crucial for the survival on *Sitodiplosis mosellana* larvae at four programmed diapause stages [17]. Similar results were also reported in diapausing eggs of Asian tiger mosquito (*Aedes albopictus*), where pathways positively associated with energy utilization are inhibited [18]. Diapausing pupae of *Rhagoletis cerasi* catabolizes a variety of lipids in summer diapause while, during the winter, lipids are not used and other resources, such as glycogen and carbohydrates, become the main sources of energy [19]. Similar patterns were observed in the flesh fly (*Sarcophaga crassipalpis*) [20] and the solitary bee (*Megachile rotundata*) [21]. By contrast, adult (reproductive) diapause is rare and less is known about its patterns of metabolite accumulation and degradation [22]. Especially, clear markers for diapause maintenance that could be used for such research design are lacking, which hindering our understanding of the diversity of diapause mechanisms.

The orange dead leaf butterfly, *Kallima inachus* (Lepidoptera: Nymphalidae) is a large-scale and well-known mimetic butterfly, is distributed in tropical and subtropical regions of eastern and southern Asia, and mainly lives in broad-leaved forests at altitudes of 500–1200 m.a.s.l [23]. In the past few decades, studies related to *K. inachus* have included research on behavior [23], biology [24], physiology [25,26], and genomics [27,28]. Due to the highly disguised wings of imagos, *K. inachus* was used as a model to demonstrate that leaf resemblance is a product of gradual evolution by natural selection [29]. Thus far, however, little information is available about metabolite changes during ontogeny in *K. inachus* although it has become primary cultivated species in southern China in recent years [30]. Understanding the development changes in metabolite levels will help in management of *K. inachus* farming. Furthermore, *K. inachus* overwinters as adults through its range in natural conditions. In the absence of predators, the female adults in diapause could live more than 316 days, whereas non-diapause females could only live about 36–59 days [31]. Several laboratory studies have been conducted to study the metabolic trends of several substances (such as trehalose and DNA) between diapause and non-diapause adults [32,33]. What is less clear currently is the metabolome of diapause individuals and the biomarkers that play an important role in diapause maintenance. Therefore, investigations into the metabolic pattern are needed to decipher the strategy for dealing with climate extremes in the females.

In the present study, 29 samples from different stages (24 larvae, pupae, adult males, adult females and five diapausing adult females) were collected. The analysis of metabolites of whole body was carried out by untargeted metabolomics based on ultra-performance liquid chromatograph coupled with tandem mass spectrometry (UPLC-MS/MS), identify main metabolites altered by the stage transitions, and address the absence of biomarkers of diapause maintenance in *K. inachus* adult females.

## 2. Materials and Methods

### 2.1. Insect Collection

About 200 disease-free eggs of orange dead leaf butterfly (*K. inachus*) were acquired from the butterfly breeding base on Leshan. The egg, larva, pupal and adult stages of experimental butterflies were reared under the natural condition (12 h/12 h light/dark cycle; ~20–25 °C; 70% relative humidity) in a net house (8 m long, 5 m wide, and 3 m tall) with evenly scattered sunlight. Larvae of *K. inachus* were reared on fresh leaves of *Strobilanthes cusia*, and the adults were fed 15% honey water. We used male and female larvae together since they were not readily distinguishable. *K. inachus* has the typical four-stage insect life cycle including egg, larval, pupal, and adult stages. After the eggs hatch, the larvae feed on the host plant, and when fully developed, pupate in a chrysalis. When metamorphosis is complete, the pupal skin splits, the adult insect climbs out. The molting time (four times) and the surficial red spines were used to identify fifth instar larvae used in this study. In this case, female adults initiate oocyte development within 4–5 days and have mature oocytes at 10–11 days after eclosion. Thus, inactive females with undeveloped oocytes on 10–11 day posteclosion were considered as individuals entering the fifth day of diapause. Samples were collected on first day of the fifth instar (LA group), fifth day after pupation (PU group), tenth day after eclosion (males, KM group; females, KF group), and fifth day after entering of diapause (female, dKF group), and six biological replicates were established. See Table 1 for the specific information of the samples. The samples were snap frozen simultaneously to limit non-biological sources of variation. Snap frozen samples were immediately stored at −80 °C.

### 2.2. Metabolite Extraction for the Untargeted Metabolomic Analysis

The sample stored at −80 °C refrigerator was thawed on ice. The thawed sample was homogenized by a grinder (30 HZ) for 20 s. A 400 μL solution (Methanol: Water = 7:3, *v*/*v*) containing internal standard was added in to 20 mg grinded sample, and shaked at 1500 rpm for 5 min. After placing on ice for 15 min, the sample was centrifuged at 12,000 rpm for 10 min (4 °C). A 300 μL of supernatant was collected and placed in −20 °C for 30 min. The sample was then centrifuged at 12,000 rpm for 3 min (4 °C). A 200 μL aliquots of supernatant were transferred for LC-MS analysis.

### 2.3. UPLC-MS/MS Analysis for the Untargeted Metabolomics

The sample extracts were analyzed using an LC-ESI-MS/MS system (HPLC, Shim-pack UFLC SHIMADZU CBM30A system, Kyoto, Japan; MS, Applied Biosystems 6500 Q TRAP, San Diego, CA, USA). The analytical conditions were as follows, UPLC: column, Waters ACQUITY UPLC HSS T3 C18 (1.8 µm, 2.1 mm × 100 mm); column temperature, 40 °C; flow rate, 0.4 mL/min; injection volume, 2 μL; solvent system, water (0.1% formic acid): acetonitrile (0.1% formic acid); gradient program, 95:5 *v*/*v* at 0 min, 10:90 *v*/*v* at 11.0 min, 10:90 *v*/*v* at 12.0 min, 95:5 *v*/*v* at 12.1 min, 95:5 *v*/*v* at 14.0 min.

LIT and triple quadrupole (QQQ) scans were acquired on a triple quadrupole-linear ion trap mass spectrometer (QTRAP), QTRAP^®^LC-MS/MS System (Applied Biosystems 4500 Q TRAP, San Diego, CA, US), equipped with an ESI Turbo Ion-Spray interface, operating in positive and negative ion mode and controlled by Analyst 1.6.3 software (AB Sciex, Framingham, US). The ESI source operation parameters were as follows: source temperature 500 °C; ion spray voltage (IS) 5500 V (positive), −4500 V (negative); ion source gas I (GSI), gas II (GSII), and curtain gas (CUR) were set at 55, 60, and 25.0 psi, respectively; the collision gas (CAD) was high. Instrument tuning and mass calibration were performed with 10 and 100 μmol/L polypropylene glycol solutions in QQQ and LIT modes, respectively. A specific set of MRM transitions were monitored for each period according to the metabolites eluted within this period. The MS RAW files for metabolite analysis have been deposited to the EMBL-EBI MetaboLights database with the identifier MTBLS5736, and can be accessed with the URL (http://www.ebi.ac.uk/metabolights/MTBLS5736 (accessed on 8 August 2022)).

### 2.4. Bioinformatic Analysis of the Untargeted Metabolomic Dataset

Unsupervised PCA (principal component analysis) was performed by statistics function prcomp within R (version 3.5.1, www.r-project.org (accessed on 25 March 2021)). The data was unit variance scaled before unsupervised PCA. In this process, dKF6 was deleted from the dKF group due to sample degradation and did not participate in the subsequent analysis. Significantly regulated metabolites between groups were determined by VIP ≥ 1 and absolute Log_2_FC (fold change) ≥ 1. VIP values were extracted from OPLS-DA result, which also contain score plots and permutation plots, was generated using R package MetaboAnalystR version 1.0.1 (McGill, QC, Canada). The data was log transform (log2) and mean centering before OPLS-DA. In order to avoid overfitting, a permutation test (200 permutations) was performed. Identified metabolites were annotated using KEGG compound database (http://www.kegg.jp/kegg/compound/ (accessed on 27 March 2021)), annotated metabolites were then mapped to KEGG pathway database (http://www.kegg.jp/kegg/pathway.html (accessed on 28 March 2021)). Significantly enriched pathways are identified with a hypergeometric test’s *p*-value for a given list of metabolites. The figure of top 10 pathways was draw using the Sangerbox tools, a free online platform for data analysis (http://vip.sangerbox.com/ (accessed on 28 March 2021)). The reliability of markers was calculated by calculating formulas as follows: R = [SD (KF)/mean (KF) + SD (dKF)/mean (dKF)].

### 2.5. Statistical Analysis

Significance of differences between groups was tested using unpaired t-tests. Correlations between variables were calculated using Pearson’s product–moment correlation. Both tests were performed using GraphPad Prism version 8.07 (GraphPad Software, La Jolla, CA, USA).

## 3. Results and Discussion

### 3.1. Untargeted Metabolite Analysis of Samples

To explore global metabolic variations, an untargeted metabolomic approach was used (*n* = 29, Table 1), which identified 1683 annotated metabolites from 3587 ion features (Table S1). Based on their annotations, plentiful metabolites were assigned to at least one primary or secondary metabolic category. The top 20 largest secondary metabolic categories, such as small peptide (191 metabolites), amino acid derivatives (85 metabolites), acyl carnitines (64 metabolites), nucleotide and its derivatives (54 metabolites), lysophosphatidyl choline (35 metabolites), and organic acid and its derivatives (33 metabolites) are shown in Figure S1.

**Figure 1:**
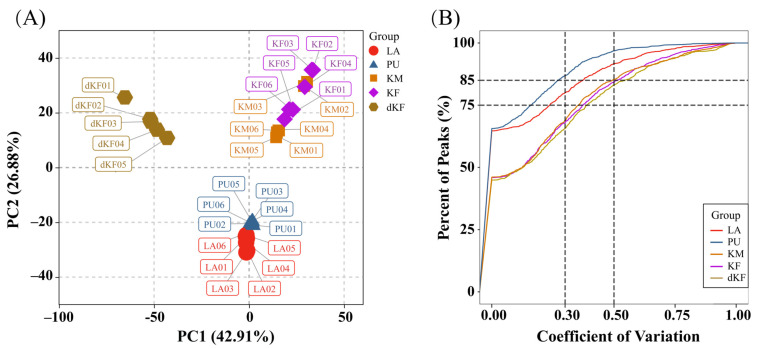
Principal component analysis (PCA) and coefficient of variation (CV) of untargeted metabolomic datasets. (**A**) PCA score plot. (**B**) coefficient of variation score plot. The x-axis indicates the CV value, and the y-axis again indicates the ratio of the number of metabolites that is less than the corresponding CV value to the total number of metabolites. Different stages of *K. inachus* are represented by different groups in different colors (larvae: LA: red; pupae: PU: blue; male adults: KM: orange; female adults: KF: purple; diapausing female adults: dKF: brown).

**Figure 2:**
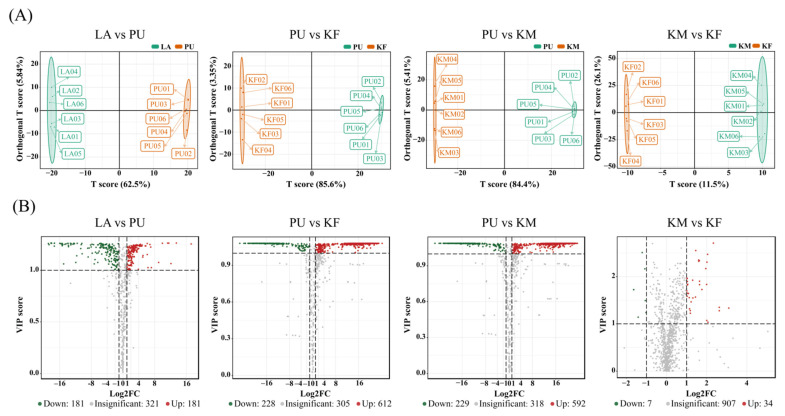
Identification of the differential accumulated metabolites (DAMs) in different comparison groups (LA vs. PU, PU vs. KF, PU vs. KM, KM vs. KF). (**A**) OPLS-DA (partial least squares discriminant analysis) score plots of four comparison groups. The x and y axes represent score for the main components in orthogonal signal correction (OSC) process (Tp) and score for the orthogonal components in OSC process (To), respectively. The abscissa and ordinate coordinates can show differences between and within groups, respectively. The percentage (%) represents the resolution of component to dataset. (**B**) The distribution of DAMs identified by the cut-off value of VIP ≥ 1 and absolute Log_2_FC ≥ 1. The green bubbles represent the significantly decreased DAMs while the red bubbles represent the significantly increased DAMs. The VIP score generated in OPLS-DA processing represented the contribution to the discrimination of each metabolite ion between groups.

To provide a deep overview of the metabolic variations, several quality control parameters for the quantification, including coefficient of variation (CV), principal component (PC) and orthogonal partial least square-discriminate analysis (OPLS-DA) were analyzed [34]. PCA score plot of these features have shown that a clear separation among different groups, with 69.8% of the variation explained by the first two principal component axes (Figure 1A, PC1 and PC2 explained 42.9% and 26.9%, respectively). To examine the quality of the acquired QC data, CV (coefficient of variation) analysis were generated for all of the samples and revealed a high degree of overlap using empirical cumulative distribution function (ECDF). From the Figure 1B we can see that the proportion of metabolites with CV value less than 0.5 in all QC samples was higher than 85%. The OPLS-DA was performed to identify the metabolites responsible for the separation between different successive stages. The score plot of OPLS-DA shows a clear separation in four comparison groups (LA vs. PU, PU vs. KM, PU vs. KF, KM vs. KF). To statistically validate the model, the permutation test (200 permutations) was performed. For LA vs. PU, R^2^X, R^2^Y, and Q^2^ were 0.685, 1.000, and 0.999, respectively; For PU vs. KM, R^2^X, R^2^Y, and Q^2^ were 0.893, 1.000, and 0.999, respectively; For PU vs. KF, R^2^X, R^2^Y, and Q^2^ were 0.883, 1.000, and 0.999, respectively; For KM vs. KF, R^2^X, R^2^Y, and Q^2^ were 0.447, 1.000, and 0.477, respectively (Figure 2A). Based on the results of OPLS-DA, 1599 high-quality metabolites were used to screen the differentially accumulated metabolites (DAMs), which were determined by variable importance in projection (VIP) ≥ 1 and absolute Log_2_FC (fold change) ≥ 1. Overall, 362 (181 up- and 181 down-regulated), 840 (612 up- and 228 down-regulated), 782 (598 up- and 298 down-regulated), and 41 (34 up- and 7 down-regulated) significant DAMs were identified in LA vs. PU, PU vs. KF, PU vs. KM, KM vs. KF, respectively (Figure 2B).

### 3.2. Vital Metabolites for Stage Development

All of the DAMs were assigned to various primary metabolic categories, including lipids, fatty acyl, heterocylic compounds, organicacid and its derivatives, oxidized lipids, carboxylic acids and derivatives, nucleotide and its metabolomics and others. The global metabolic captured the 120 DAMs with significant difference in two transitions of successive stages (larva to pupa and pupa to adult).

**Figure 3:**
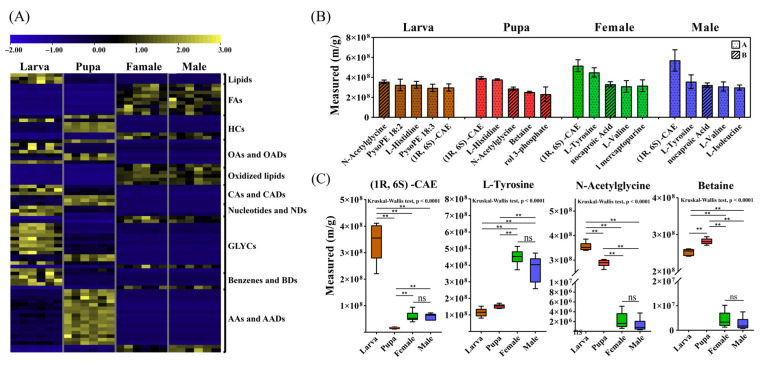
The overview of metabolomes of different developmental stages. (**A**) Heat map of 120 DAMs with significant difference in two transitions of successive stages (larva to pupa and pupa to adult). The level of metabolite increases from blue to yellow. (**B**) The top five small-molecule chemicals (heavyweights) with the highest content at each stage, respectively. The level of substance identification was indicated by letters. The ‘A’ and ‘B’ represent ion fragments of two and one metabolites detected, respectively. (**C**) Four metabolites that remains high content throughout ontogeny. Statistical significance is indicated as asterisks in figures as (ns) for *p* > 0.05, (**) for *p* < 0.01. Different stages of *K. inachus* are represented by different colors (larvae: brown; pupae: red; male adults: blue; female adults: green).

As shown in Figure 3A, plenty of glycerophospholipids 11 metabolites such as lysope 18:0, pysoPE 20:2, and pysoPE 18:2 were predominantly accumulated in larval stage, while several carboxylic acids or its derivatives (three metabolites), nucleotide or its metabolomics (three metabolites), and benzene and substituted derivatives (five metabolites) have higher levels in larva than other stages. For pupal stage, the most striking characterization is abundant amino acid and its derivatives (16 metabolites, such as L-tryptophan and L-glutamate) which did not maintain in the former or the latter stages, while the levels of four heterocyclic compounds peak in this stage. For ault stage, in addition to the sharp drop in the levels of various amino acid or its metabolomics after pupal stage, the most notable was the large accumulation of multiple fatty acyls (nine metabolites). Furthermore, there were no significant differences in vital metabolic categories for transition of pupal to adult stages between males and females.

### 3.3. Vital Metabolites for Transition of Successive Stages

Table 2 shows the top five DAMs (up- or down) identified based on log_2_FC in transition of successive stages and their related metabolic pathways. In LA vs. PU, 2-Keto-6-acetamidocaproate, carnitine C13:1, N-phenylacetylglycine, 3-Chloro-L-tyrosine, and carnitine C14:1-OH are the DAMs with the maximum Log_2_FC value; Cinnabarinic acid, trans, cis-3,6-nonadien-1-ol, 2-(formylamino) benzoic acid, 13,14-Dihydro-15-keto-PGD2, and (Z)-6-octadecenoic acid are the DAMs with the minimum Log_2_FC value. In PU vs. KF (stands for imagoes), DL-O-tyrosine, tauropine, L-palmitoylcarnitine, carnitine C16:0, and L-histidine are the DAMs with the maximum Log_2_FC value; L-glutamate, L-glutamine, ®-(-)-2-phenylglycine, L-isoisoleucine, and L-tryptophan are the DAMs with the minimum Log_2_FC value. In KM vs. KF, 4-O-Dimethylallyl-L-tyrosine, N-lactoyl-phenylalanine, Salicin-6P, Uridine triphosphate, and 3-Hydroxypicolinic acid are the DAMs with the maximum Log_2_FC value; 5’-deoxyadenosine, 2’-deoxyadenosine, S-Sulfo-L-cysteine, protocatechuic aldehyde, carnitine C9:2-OH are the DAMs with the minimum Log_2_FC value.

### 3.4. The Endogenous Metabolome of Different Developmental Stages

The endogenous metabolome is the set of endogenous metabolites in biological samples, which can reflect the endogenous mechanisms of adaptation and the small-molecule chemicals that play a role in survival within species [35]. In this study, the endogenous metabolome of larval, pupal, and adult (males and females) stages in *K. inachus* were generated by LC-MS. The top five small-molecule chemicals (heavyweights) with the highest content at each stage were significantly different between immature and mature stages (Figure 3B). After searching the metabolite pool of LA groups, N-acetylglycine, pysoPE 18:2, L-histidine, pysoPE 18:3, and (1R, 6S)-6-amino-5-oxocyclohexyl-2-ene-1-carboxylic acid ester (CAE) were identified as heavyweights in larval stage. When entering the pupal stage, three heavyweights ((1R, 6S)-CAE, L-histidine, and N-acetylglycine) in larval stage were retained but their ranking changed. Two new metabolites (betaine and Glycerol-3-phosphate) were identified as heavyweights in this stage. In adult stage, the top four metabolites ((1R, 6S) -CAE, L-tyrosine, 6-aminocaproic acid, and L-valine) in both males and females are identical, while the fifth heavyweight are 6-Methyl mercaptopurine in females and L-isoleucine in males. The bar graph above shows the major characteristic of the endogenous metabolome is the high assay of (1R, 6S) -CAE during ontogeny in *K. inachus* (Figure 3C). The content of L-tyrosine was the second in adult stage. Furthermore, the average molecular weight of heavyweights gradually decreases as life cycle progresses (284.74, 149.25, 144.06, and 143.08 Da in larvae, pupae, females, and males, Table S2).

### 3.5. Diapause-Associated Changes in Metabolite Profiles

**Figure 4:**
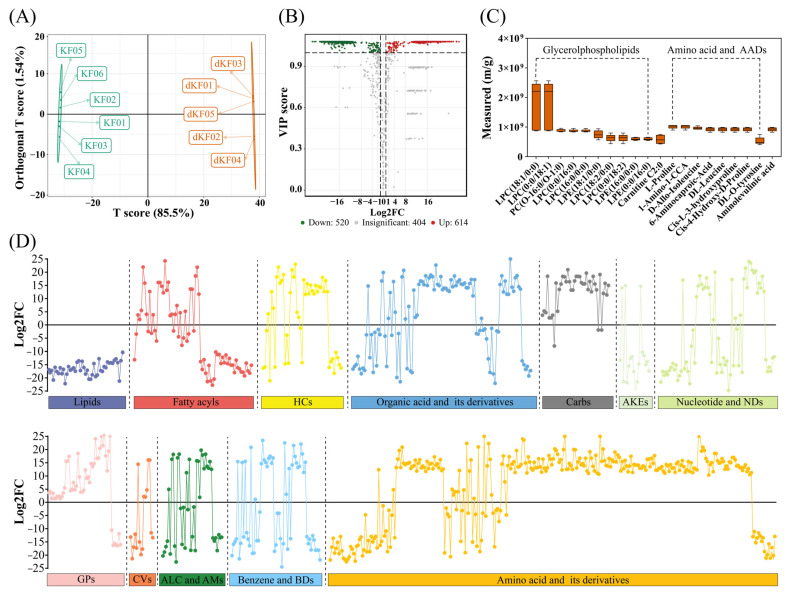
Metabolome analysis between non-diapause (KF) and diapause (dKF) adult females. (**A**) OPLS-DA score plots. (**B**) The distribution of DAMs identified by the cut-off value of VIP ≥ 1 and absolute Log2FC ≥ 1. (**C**) The top 20 metabolites with the highest content at diapause individuals. (**D**) The metabolic profiling of diapausing female adult based on Log_2_FC. The x-axis represents different primary classification of metabolites which differentiated by different colors.

The aforementioned analyses provide overall changes in metabolite profiles during ontogeny in *K. inachus*. The next part of this study was concerned on the biomarkers of entering the diapause maintenance and main metabolic pathways enriched by DAMs identified between KF and dKF groups. The metabolic pools of KF and dKF were compared in order to study the difference between the metabolome in diapause and non-diapause states of *K. inachus* adult female. The score plot of OPLS-DA shows a clear separation of diapause and non-diapause samples (Figure 4A). To statistically validate the model, the permutation test (200 permutations) was performed. The R^2^X and R^2^Y of the OPLS-DA model was 0.87 and 1.00, indicating the explanatory rate of the model to the X and Y matrix is high. The Q^2^ was 0.999, indicating a good predictivity. Our untargeted metabolomics dataset (Figure 4B) included 614 metabolites that were less abundant (log_2_FC ≤ −1, *p* ≤ 0.05) and 520 metabolites that were more abundant (log_2_FC ≥ 1, *p* ≤ 0.05) in diapause adult females relative to non-diapause adult females. The top 20 heavyweights with the highest content at diapause individuals were highly taxonomically concentrated. Among them, 50% of the heavyweights were glycerophospholipids and 45% were amino acids or AADs (Figure 4C). Further analysis showed that more than 90% of DAMs could be classified into 12 primary categories in main metabolite profiles (Figure 4D).

Several DAMs were identified as potential biomarkers of entering the diapause maintenance based on the ®io (R) of standard deviation to mean in two groups. Several representative biomarkers belong to glycerophospholipids and amino acids or AADs are presented in Table 3.

### 3.6. Key Pathways Affected by Entering into Adult Diapause

**Figure 5:**
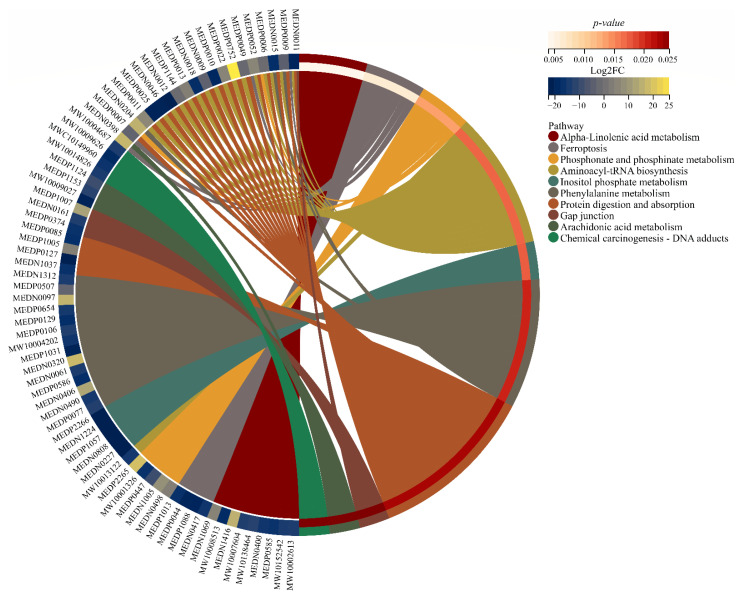
The circle plot of enriched KEGG pathways for DAMs in KF vs. dKF. The Log_2_FC of DEGs in the corresponding pathway was represented by color from blue to yellow (low to high), and the enrichment *p*-value of the corresponding pathway was represented by the colors from white to red (low to high). MEDxxx, MWxxx or MWCxxx are the IDs of identified metabolites in the database. The connecting lines represent metabolites (left semicircle) are enriched to the KEGG pathway (right semicircle).

Through the KEGG pathway analysis of the DAMs, the variational metabolic pathways caused by entering diapause maintenance in *K. inachus* adult females were analyzed. The enrichment of pathways is presented in Figure 5. Over 300 metabolites across more than 20 pathways, including the metabolism of lipids, amino acids, carbohydrates and coenzymes were changed in diapause individuals. Detailed enriched pathway information is provided in Table S3. L-glutamic acid (MEDN0011), L-phenylalanine (MEDN0015), L-tyrosine (MEDP0009), L-glycine (MEDP0006), and L-cysteine (MEDP0052), etc., are the metabolites enriched to multi-pathways, suggesting that the changes of major metabolites during diapause in *K. inachus* are complex and multi-pathway cooperative. Detailed metabolite information is provided in Table S1.

## 4. Discussion

The mechanism of complete metamorphosis is one of the most important challenges in the study of holometabolism. However, the metabolite changes during stage transitions have not been systematically studied in holometabolous insects. Similarly, data on the accumulation and degradation of metabolites during adult diapause are scarce, and the lack of biomarkers make it difficult to identify diapause adults occurring in natural state. Therefore, in this study we identified vital metabolites for stage development and transition of successive stages in *K. inachus*. Among the four comparable groups (LA vs. PU, PU vs. KF, PU vs. KM, and KF vs. KM), there were more DAMs in the PU vs. KM (782) or KF (840) than in the LA vs. PU comparisons (362), indicating that eclosion of *K. inachus* pupae results in expression changes of many DAMs although pupation dramatically changes the insect’s appearance physiology and functional structure [36]. The reason for this phenomenon is likely to be the intensity of the endocrine system affected by microRNA varies between pupation (high) and eclosion (low) [37].

For larval stage development, 11 glycerophospholipids (seven lysophosphatidylcholines and three lysophosphatidylethanolamines) were found to accumulate specifically in the larval stage of *K. inachus*. Moderate accumulation of glycerophospholipids has also been observed in first-instar of other holometabolic insects [38]. Glycerophospholipids are the main component of biological membranes. The glycerophospholipids metabolism was found to be related to the lipid regulation, lipoprotein, whole-body energy metabolism and metabolic disorders [39]. Cellular lipids have been reported to play crucial roles in the replication of a number of viruses, including enveloped viruses and no-enveloped viruses [40]. This may be the metabolic basis of the larval stage as the susceptibility stage of nuclear polyhedrosis virus (NPV) in Lepidoptera. For pupal stage development, a variety of common amino acids specifically accumulate at this stage. Among them, L-tryptophan and L-glutamate may be more important for the chrysalis. L-tryptophan is a heterocyclic amino acid and the major limiting amino acids in most insects [41]. In the majority of holometabolous insects, most larval tissues and organs are actually re-specified, including the epidermis, nervous system and muscles [3]. It has been proved that the determinant of pupal viability of D. melanogaster cinnabar and cardinal eye color mutants is tryptophan metabolism [42]. Therefore, high levels of L-tryptophan may be associated with characteristic formation and eclosion during pupal stage of *K. inachus*. Notably, after about 20 days, tryptophan levels dropped to almost undetectable levels in imagoes. An acceptable assumption is that tryptophan is converted to nicotinic acid for preservation in adults, which is supported by metabolome data showing a 6-fold increase (*p* < 0.05) in nicotinic acid from pupal to adult stages (Table S1). In addition, prior studies that have noted the tryptophan was used to synthesize pigment in butterflies such as the papiliochromes [43]. This reference may support the hypothesis that tryptophan as precursors of ‘supposed’ pigment, which is indispensable to the formation of wing patterns of *K. inachus*. One unanticipated finding was the accumulation of L-glutamate in pupal stage because studies in fruit fly have found that L-glutamate is inactive in the pupal and larval stages [44]. L-glutamate mediates fast inhibitory neurotransmission by affecting glutamate-gated chloride channels in invertebrates [45]. Hence, the reason of immobile and slow-acting in chrysalis of *K. inachus* maybe fast inhibitory neurotransmission caused by the high content of L-glutamate. For adult stage development, levels of several free fatty acids were found to increase dramatically. Free fatty acids are the product of lipolysis of storage fat, i.e., triacylglyceroles [46]. As mentioned in the study of *S. argyrostoma*, the free fatty acids profile varies considerably between each development stage [47].

Fold change analysis showed that the drastic changes in the concentrations of the following four DAMs may be the trigger for pupation. (1) 2-Keto-6-acetamidocaproate is the intermediate of L-lysine to L-alpha-aminoadipate and may have the potential detoxification abilities to protect the *K. inachus* during pupal stage [48]. (2) N-phenylacetylglycine was found to scavenge extracellular peroxide (pyruvate), inhibit ROS and activate cellular antioxidant defense, act as indicators of antioxidant mobilization against oxidative stress [49]. (3) Cinnabarinic acid is an endogenous metabolite of the kynurenine pathway that meets the structural requirements to interact with glutamate receptor, [50]. (4) 2-(Formylamino) benzoic acid is an upstream metabolite of cinnabarinic acid, and the decrease of both is to accumulate L-kynurenine which promoter the degradation of tryptophan, and biosynthesis eye chromes to protect nervous system in insects (Log_2_FC = 3.50, *p* < 0.05) [51]. Three DAMs (including increased L-histidine and decreased L-glutamate and L-glutamine) related to histidine metabolism were identified in the transition of pupal to adult stages. Recent study showed that Lepidopteran wing scales contain abundant histidine-rich cuticular proteins [52]. This is one of the reasons why biosynthesis of histidine was promoted in adults. The regulation of histidine metabolism is the key to eclosion. Several DAMs related to nucleotide and amino acid metabolism have also been found between males and females. Therefore, we hypothesize that this phenomenon is due to female oogenesis earlier than male spermatogenesis. The function of the most of remaining top DAMs in insects remains unclear. However, it is necessary to study these metabolites in further study because of the great change of their levels during the stage transitions.

(1R, 6S) -CAE is a metabolin in phenazine biosynthes and an intermediate from chorismate to phenazine [53]. In this study, (1R, 6S) -CAE is the 1st metabolite in the adult and pupal stages but the fifth in the larval stage in content. Actually, the content of (1R, 6S) -CAE in larvae is 3–5 times (*p* < 0.05) that in other stages. However, the reason for the high level of (1R, 6S) -CAE in all developmental stages, especially larval stage, and its evolutionary background are still unclear. Large accumulations of this metabolite are not seen in other holometabolous insects until now. In contrast to (1R, 6S) -CAE, further statistical test revealed that L-tyrosine in adult is 3–5 times (*p* < 0.05) that in other stages. L-tyrosine is the substrates for melanin biosynthesis, which have been demonstrated to regulate color and morphology of butterfly wing scales [54]. The other two metabolites with high concentrations are N-acetylglycine and betaine, which were significantly different (*p* < 0.05) between larval and pupal stages. However, this difference is much smaller than the decline after entering the mature stage.

Furthermore, we also plotted the major metabolic profiles during diapause in *K. inachus* adult females and provided several potential biomarkers of diapause maintenance. Surprisingly, all DAMs belong to lipids were decreased in the dKF group, but most DAMs belong to GPs were detected to be highly up-regulated. This finding is consistent with that of R. cerasi diapause pupae which catabolize a variety of lipids during the summer diapause [19]. Meanwhile, female adults accomplished seasonal acquisition of chill tolerance by synthesizing large amounts of glycerophospholipids that are different from those accumulated during larval stage. This result is in line with those of previous study on *P. apterus* [55]. Similar to the case of glycerophospholipids, the level of most members of carbohydrates (carbs) DAMs was also found to been deeply raised. Increasing carbohydrate content is one of the strategies used by many insects to combat extreme weather conditions in both pupal and larval diapauses [56,57,58]. Combined with the results of this study, the increase of carbohydrate content is an important marker of early diapause maintenance in all stages in insects. Further, several reports have showed the importance of trehalose during diapause in invertebrates [59,60]. In present study, metabolome of dKF matched those observed in earlier studies of Yi et al. [33] who concluded the type of carbohydrate-alcohol accumulation of *K.inachus* was trehalose. For the category with the most detected DAMs, amino acids and AADs, the level changes of DAMs are slightly complicated but still most DAMs also rose steadily. Amino acids and their derivatives vary greatly during egg [61], larval [62] or adult [63] diapause. This discrepancy could be attributed to the metabolic system of amino acids to adapt to the needs of biosynthesis and environmental stress. In conclusion, DAMs belong to glycerophospholipids, carbs and amino acids and AADs in this study are suitable as detectable biomarkers for entering the diapause maintenance stage of adults.

KEGG enrichment analysis indicate several pathways are associated with adult diapause in *K. inachus* females. Regulating these targeted pathways may achieve purposeful intervention in the expression of metabolites. Most of the pathways enriched only accumulated one or two metabolites (converted from other metabolites of the pathway or from upstream pathways) in dKF groups. For example, in alpha-linolenic acid metabolism, the level of 9(S)-HOTrE increases with the decreasing level of alpha-linolenate, 13(S)-HpOTrE and others. 9(S)-HOTrE is an octadecanoid that could increase apolipoprotein transcription [64]. Active lipid metabolism could be observed in the metabolic profile above. A possible explanation for this might be that accumulation of 9(S)-HOTrE in diapause adults promotes the production of apolipoprotein and then regulates active lipid metabolism. In further study, we will investigate the differences in physical index (such as cold tolerance, lifespan, and activeness), transcriptome and methylome between the second and third generations of *K. inachus* which are lipoxygenase (a key enzyme of 9(S)-HOTrE) knockdown through transcriptome and whole genome bisulfite sequencing. Ferroptosis is a new type of cell death that was discovered in recent years and is usually accompanied by a large amount of iron accumulation and lipid peroxidation during the cell death process [65]. Larval-specific tissues (such as prothoracic glands and anterior silk glands) are normally eliminated by programmed cell death after pupation [66]. However, for imagoes, the accumulation of cysteine and arachidonate acid through Ferroptosis is likely to be the key to entering into diapause [67]. At present, there are few studies on alpha-linolenic acid metabolism and ferroptosis in diapause insects. The results can provide a reference for relevant studies of targeted pathways.

## 5. Conclusions

In this study, we investigated the metabolic changes in *K. inachus* during ontogeny and diapause by untargeted metabolomics (UPLC-MS/MS), analyzed the vital metabolites for stage development and transition of successive stages, and summarized potential biomarkers for entering into diapause stage of adults and key pathways affected by entering into adult diapause.

Glycerophospholipids, amino acid or its derivatives, and fatty acyls is crucial for larval, pupal, and adult stages of *K. inachus*, respectively. 2-keto-6-acetamidocaproate, N-phenylacetylglycine, Cinnabarinic acid, and 2-(Formylamino) benzoic acid are momentous in stage transition of larva to pupa; L-histidine, L-glutamate and L-glutamine are momentous in stage transition of pupa to imago; Levels of (1R, 6S) -CAE and L-tyrosine are high throughout development but the specific functions still need to be explored. Lipids degradation and synthesis of glycerophospholipids and carbohydrates were observed in diapause individuals, and the metabolism of amino acids and their derivatives was very active in diapause individuals. Alpha-linolenic acid metabolism and ferroptosis were first found to be associated with diapause in adults. Our study lays the foundation for a systematic study of the developmental mechanism of holometabolous insects and metabolic basis of adult diapause in butterflies.

## Figures and Tables

**Table 1:** Information of samples used for untargeted metabolomic analysis.

Group	Members	Treatment	Collection Time	State
LA	LA01 (Ki084), LA02 (Ki022), LA03 (Ki025), LA04 (Ki050), LA05 (Ki084), LA06 (Ki113)	liquid nitrogen	2020.5.30–2020.6.14	first day of the fifth instar
PU	PU01 (Ki007), PU02 (Ki012), PU03 (Ki129), PU04 (Ki067), PU05 (Ki047), PU06 (Ki081)	liquid nitrogen	2020.6.17–2020.6.28	fifth day after pupation
KM	KM01 (Ki040), KM02 (Ki018), KM03 (Ki080), KM04 (Ki035), KM05 (Ki189), KM06 (Ki159)	liquid nitrogen	2020.7.03–2020.7.22	tenth day after eclosion (males)
KF	KF01 (Ki026), KF02 (Ki129), KF03 (Ki022), KF04 (Ki015), KF05 (Ki031), KF06 (Ki078)	liquid nitrogen	2020.7.06–2020.7.29	tenth day after eclosion (females)
dKF	dKF01 (Ki032), dKF02 (Ki116), dKF03 (Ki115), dKF04 (Ki058), dKF05 (Ki063), dKF06 (Ki066)	liquid nitrogen	2021.2.23–2021.2.24	fifth day after entering of diapause (females)

Different groups (LA, PU, KM, KF, dKF) represent different stages (larvae, pupae, male adults, female adults and diapausing female adults) of *K. inachus*.

**Table 2:** The top five DAMs (up- or down) identified based on log_2_FC in transition of stages.

Name	Formula	Primary Category	VIP	*p*-Value	Log_2_FC	Pathway
LA vs. PU (up)						
2-Keto-6-acetamidocaproate	C_8_H_13_NO_4_	-	1.25	9.21 × 10^−^^3^	17.25	Lysine degradation
Carnitine C13:1	C_20_H_37_NO_4_	Lipids	1.26	8.67 × 10^−^^5^	13.49	-
N-phenylacetylglycine	C_10_H_11_NO_3_	AAD	1.06	2.31 × 10^−^^2^	12.14	Phenylalanine metabolism
3-Chloro-L-tyrosine	C_9_H_10_C_l_NO_3_	AAD	1.26	1.46 × 10^−^^4^	11.59	-
Carnitine C14:1-OH	C_21_H_39_NO_5_	Lipids	1.26	8.83 × 10^−^^5^	11.30	-
LA vs. PU (down)						
Cinnabarinic acid	C_14_H_8_N_2_O_6_	AAD	1.25	7.10 × 10^−^^3^	−18.84	Tryptophan metabolism
Trans,cis-3,6-Nonadien-1-ol	C_9_H_16_O	Alcohol	1.25	1.60 × 10^−^^2^	−18.68	-
2-(Formylamino) benzoic acid	C_8_H_7_NO_3_	BD	1.26	1.24 × 10^−^^4^	−18.20	Tryptophan metabolism
13,14-Dihydro-15-keto-PGD2	C_20_H_32_O_5_	Lipids	1.26	4.04 × 10^−^^5^	−18.07	-
(Z)-6-octadecenoic acid	C_18_H_34_O_2_	Lipids	1.26	7.51 × 10^−^^3^	−18.05	-
PU vs. KF (up)						
DL-O-tyrosine	C_9_H_11_NO_2_	AAD	1.08	1.73 × 10^−^^3^	24.21	-
Tauropine	C_5_H_11_NO_5_S	AAD	1.09	3.48 × 10^−^^6^	23.03	-
L-palmitoylcarnitine	C_25_H_49_NO_4_	FA	1.09	1.87 × 10^−^^4^	22.96	Fatty acid metabolism
Carnitine C16:0	C_23_H_45_NO_4_	FA	1.08	1.26 × 10^−^^3^	22.74	Fatty acid metabolism
L-histidine	C_6_H_9_N_3_O_2_	Amino acids	1.09	1.31 × 10^−^^5^	22.52	Histidine metabolism
PU vs. KF (down)						
L-glutamate	C_5_H_9_NO_4_	Amino acids	1.08	1.41 × 10^−^^5^	−23.91	Histidine metabolism
®(R)-(-)-2-phenylglycine	C_8_H_11_NO	ACD	1.08	9.26 × 10^−^^4^	−23.37	-
L-isoisoleucine	C_6_H_13_NO_2_	ACD	1.08	1.71 × 10^−^^2^	−22.48	-
L-tryptophan	C_11_H_12_N_2_O_2_	ACD	1.08	1.89 × 10^−^^2^	−22.36	Tryptophan metabolism
KM vs. KF (up)						
4-O-Dimethylallyl-L-tyrosine	C_14_H_19_NO_3_	AAD	1.33	1.76 × 10^−^^2^	3.10	-
N-lactoyl-phenylalanine	C_12_H_15_NO_4_	AAD	1.28	4.04 × 10^−^^2^	2.63	-
Salicin-6P	C_13_H_19_O_10_P	Glycosides	1.35	4.04 × 10^−^^2^	2.62	Gly/Glu
Uridine triphosphate	C_9_H_15_N_2_O_15_P3	ND	2.71	5.04 × 10^−^^3^	2.33	Pyrimidine metabolism
3-hydroxypicolinic acid	C6H5NO3	HC	1.03	4.19 × 10^−^^2^	2.06	-
KM vs. KF (down)						
5’-deoxyadenosine	C_10_H_13_N_5_O_3_	ND	1.72	3.22 × 10^−^^2^	−1.64	-
2’-deoxyadenosine	C_10_H_13_N_5_O_3_	ND	1.72	2.09 × 10^−^^2^	−1.63	Purine metabolism
S-Sulfo-L-xysteine	C_3_H_7_NO_5_S_2_	AAD	1.14	2.88 × 10^−^^2^	−1.41	Cys and Met metabolism
Protocatechuic aldehyde	C_7_H_6_O_3_	BD	2.51	1.41 × 10^−^^3^	−1.21	-
Carnitine C9:2-OH	C_16_H_27_NO_5_	FA	2.17	3.62 × 10^−^^2^	−1.08	-

A metabolite with a high VIP score contributes more to the difference between groups. *p*-value < 0.05 was used as the statistical significance threshold. Log_2_FC (foldchange) can reveal the multiple of metabolite change from the control to the experimental groups. Pathway information was derived from KEGG enrichment analysis and indicates the role of metabolite in biological metabolism and cell signal transduction.

**Table 3:** Potential biomarkers (glycerophospholipids and amino acids or AADs) for entering the diapause maintenance stage of adults.

Name	Formula	Second Category	In Diapause Adult Females	Reliability
Glycerophospholipids				
LPC(13:0/0:0)	C_21_H_44_NO_7_P	lysophosphatidylcholine	Increase	High
PC(8:0/8:0)	C_24_H_48_NO_8_P	phosphatidylcholine	Increase	Relatively high
LPE(17:1/0:0)	C_22_H_44_NO_7_P	Lysophosphatidylethanolamine	Increase	High
LPA(16:0/0:0)	C_19_H_39_O_7_P	lysophosphatidic acid	Increase	High
LPS(22:6/0:0)	C_28_H_44_NO_9_P	Lysophosphatidylserine	Increase	Relatively high
PysoPS 18:2	C_24_H_44_NO_9_P	Lysophosphatidylserine	Decrease	High
Lysopg 18:1	C_24_H_47_O_9_P	Lysophosphatidyl glycerol	Decrease	Moderate
PI 18:3	C_27_H_47_O_12_P	phosphatidyl inositol	Decrease	Relatively high
Amino acids and AADs				
L-alanine	C_3_H_7_NO_2_	Amino acids	Increase	Moderate
L-cysteine	C_3_H_7_NO_2_S	Amino acids	Increase	Relatively high
L-proline	C_5_H_9_NO_2_	Amino acids	Increase	Relatively high
L-theanine	C_7_H_14_N_2_O_3_	Amino acids	Increase	High
L-arginine	C_6_H_14_N_4_O_2_	Amino acids	Decrease	Relatively high
Cis-L-3-hydroxyproline	C_5_H_9_NO_3_	Amino acid derivatives	Increase	High
L-threo-3-methylaspartate	C_5_H_9_NO_4_	Amino acid derivatives	Increase	High
L-pyroglutamic acid	C_5_H_7_NO_3_	Amino acid derivatives	Increase	Relatively high
Glutathione	C_20_H_32_N_6_O_12_S_2_	Small peptides	Decrease	High
Glutathione reducedform	C_10_H_17_N_3_O_6_S	Small peptides	Decrease	High
N-Acetyl-Asp-Glu	C_11_H_16_N_2_O_8_	Small peptides	Increase	Moderate

The R value corresponds to the reliability level as follows: R ≤ 0.1, reliability: High; 0.1 < R ≤ 0.2, reliability: Relatively high; 0.2 < R ≤ 0.3, reliability: Moderate.

## Data Availability

The data presented in this study are available in article and supplementary material.

## Supplementary Materials

The following supporting information can be downloaded at: https://www.mdpi.com/article/10.3390/metabo12090804/s1. Figure S1: The top 20 largest metabolic secondary categories of all identified metabolites. The Y-axis represents the number of metabolites. Table S1: Detailed information of the metabolites identified in untargeted metabolome, Table S2: The molecular weight of top five small-molecule chemicals with the highest content. Table S3. Detailed information of the KEGG pathway enriched by DAMs identified between KF and dKF groups.

## Abbreviations

TCA	Tricarboxylic acid
VIP	Variable importance in projection
AA	Amino acid
AAD	Amino acid derivative
BD	Benzene derivative
FA	Fatty acyl
OA	Organic acid
OAD	Organic acid derivative
CA	Carboxylic acid
CAD	Carboxylic acids derivative
GLYC	Glycerophospholipid
Gly/Glu	Glycolysis/Gluconeogenesis
ND	Nucleotide derivative
HC	Heterocyclic compound
Cys and Met	Cysteine and methionine
AKEs	Aldehyde, Ketones, Esters
CVs	CoEnzyme and vitamins
ALC and AMs	Alcohol and amines

## References

[1] Labandeira C.C., Phillips T.L. (1996). A carboniferous insect gall: Insight into early ecologic history of the Holometabola. Proc. Natl. Acad. Sci. USA.

[2] Hammer T.J., Moran N.A. (2019). Links between metamorphosis and symbiosis in holometabolous insects. Philos. Trans. R. Soc. Lond. B Biol. Sci..

[3] Rolff J., Johnston P.R., Reynolds S. (2019). Complete metamorphosis of insects. Philos. Trans. R. Soc. Lond. B Biol. Sci..

[4] Duneau D.F., Lazzaro B.P. (2018). Persistence of an extracellular systemic infection across metamorphosis in a holometabolous insect. Biol. Lett..

[5] Snart C.J., Hardy I.C., Barrett D.A. (2015). Entometabolomics: Applications of modern analytical techniques to insect studies. Entomol. Exp. Appl..

[6] Zhang A., Sun H., Wang P., Han Y., Wang X. (2012). Modern analytical techniques in metabolomics analysis. Analyst.

[7] Phalaraksh C., Reynolds S.E., Wilson I.D., Lenz E.M., Nicholson J.K., Lindon J.C. (2008). A metabonomic analysis of insect development: ^1^H-NMR spectroscopic characterization of changes in the composition of the haemolymph of larvae and pupae of the tobacco hornworm. Manduca Sexta. Sci..

[8] Zhou L., Li H., Hao F., Li N., Liu X., Wang G., Wang Y., Tang H. (2015). Developmental changes for the hemolymph metabolome of silkworm (*Bombyx mori* L.). J. Proteome. Res..

[9] Cao Y.Y., Peng L.L., Jiang L., Thakur K., Hu F., Tang S.M., Wei Z.J. (2020). Evaluation of the metabolic effects of hydrogen sulfide on the development of *Bombyx mori* (Lepidoptera: Bombycidae), using liquid chromatography-mass spectrometry-based metabolomics. J. Insect. Sci..

[10] Chowdhury S., Fuller R.A., Dingle H., Chapman J.W., Zalucki M.P. (2021). Migration in butterflies: A global overview. Biol. Rev. Camb. Philos. Soc..

[11] Radchuk V., Turlure C., Schtickzelle N. (2013). Each life stage matters: The importance of assessing the response to climate change over the complete life cycle in butterflies. J. Anim. Ecol..

[12] Posledovich D., Toftegaard T., Wiklund C., Ehrlén J., Gotthard K. (2015). Latitudinal variation in diapause duration and post-winter development in two pierid butterflies in relation to phenological specialization. Oecologia.

[13] Ichikawa T., Aoki S., Shimizu I. (1997). Neuroendocrine control of diapause hormone secretion in the silkworm. Bombyx Mori. J. Insect Physiol..

[14] Emerson K.J., Bradshaw W.E., Holzapfel C.M. (2009). Complications of complexity: Integrating environmental, genetic and hormonal control of insect diapause. Trends Genet..

[15] Lehmann P., Pruisscher P., Koštál V., Moos M., Šimek P., Nylin S., Agren R., Väremo L., Wiklund C., Wheat C.W. (2018). Metabolome dynamics of diapause in the butterfly *Pieris napi*: Distinguishing maintenance, termination and post-diapause phases. J. Exp. Biol..

[16] Mikucki E.E., Lockwood B.L. (2021). Local thermal environment and warming influence supercooling and drive widespread shifts in the metabolome of diapausing *Pieris rapae* butterflies. J. Exp. Biol..

[17] Huang Q., Ma Q., Li F., Zhu-Salzman K., Cheng W. (2022). Metabolomics reveals changes in metabolite profiles among pre-diapause, diapause and post-diapause larvae of *Sitodiplosis mosellana* (Diptera: Cecidomyiidae). Insects.

[18] Batz Z.A., Armbruster P.A. (2018). Diapause-associated changes in the lipid and metabolite profiles of the asian tiger mosquito, *Aedes albopictus*. J. Exp. Biol..

[19] Papanastasiou S.A., Nestel D., Diamantidis A.D., Nakas C.T., Papadopoulos N.T. (2011). Physiological and biological patterns of a highland and a coastal population of the European cherry fruit fly during diapause. J. Insect Physiol..

[20] Michaud M.R., Denlinger D.L. (2006). Oleic acid is elevated in cell membranes during rapid cold-hardening and pupal diapause in the flesh fly, *Sarcophaga crassipalpis*. J. Insect Physiol..

[21] Yocum G.D., Kemp W.P., Bosch J., Knoblett J.N. (2005). Temporal variation in overwintering gene expression and respiration in the solitary bee *Megachile rotundata*. J. Insect Physiol..

[22] Hiroyoshi S., Reddy G.V.P. (2018). Field and laboratory studies on the ecology, reproduction, and adult diapause of the asian comma butterfly, *Polygonia c-aureum* L. (Lepidoptera: Nymphalidae). Insects.

[23] Tang Y., Zhou C., Chen X., Zheng H. (2013). Foraging behavior of the dead leaf butterfly, *Kallima inachus*. J. Insect Sci..

[24] Wan W.T., Dong Z.W., Ren Y.D., Yang J., Pan X.Y., He J.W., Chang Z., Liu W., Liu G.C., Zhao R.P. (2021). Chromatin accessibility profiling provides insights into larval cuticle color and adult longevity in butterflies. Zool. Res..

[25] Fei Y.H., Yang J.T. (2016). Importance of body rotation during the flight of a butterfly. Phys. Rev. E.

[26] Suzuki T.K., Tomita S., Sezutsu H. (2019). Multicomponent structures in camouflage and mimicry in butterfly wing patterns. J. Morphol..

[27] Qin X.M., Guan Q.X., Zeng D.L., Qin F., Li H.M. (2012). Complete mitochondrial genome of *Kallima inachus* (Lepidoptera: Nymphalidae: Nymphalinae): Comparison of *K. inachus* and *Argynnis hyperbius*. Mitochondr. DNA.

[28] Yang J., Wan W., Xie M., Mao J., Dong Z., Lu S., He J., Xie F., Liu G., Dai X. (2020). Chromosome-level reference genome assembly and gene editing of the dead-leaf butterfly *Kallima inachus*. Mol. Ecol. Resour..

[29] Watson D., Timofeeff-Ressovsky N.W., Salisbury E.J., Turrill W.B., Jenkin T.J., Ruggles Gates R., Fisher R.A., Diver C., Hale Carpenter G.D., Haldane J.B.S. (1936). A Discussion on the present state of the theory of natural selection. Pro. R. Soc. Lond. B Biol. Sci..

[30] Li J. (2019). A Study on Characteristics of Reproductive Dormancy in Overwintering Kallima inachus.

[31] Zhou C.L. (2008). Studies on the Biological Deatures and Molecular Intraspecific Fenetic Differentiation of Kallima inachus (Lepidoptera: Nymphalidae).

[32] Yi C.H., Chen X.M., Shi J.Y., Zhou C.L. (2009). Change of nucleic acid contents in the non-diapause and over-winter adult of *Kallima inachus*. Shandong For. Sci. Technol..

[33] Yi C.H., Chen X.M., Shi J.Y., Zhou C.L. (2009). Change of carbohydrate content in the non-diapause and over-winter adult of *Kallima inachus*. Shandong For. Sci. Technol..

[34] Wu C., Wang H., Liu Z., Xu B., Li Z., Song P., Chao Z. (2022). Untargeted metabolomics coupled with chemometrics for leaves and stem barks of dioecious *Morus alba* L. Metabolites.

[35] Liu J.Y., Zheng R.Q., Wang Y., Liu Y.H., Jiang S., Wang X.Z., He K., Pan X., Zhou T., Li T. (2022). The endogenous metabolite glycerophosphocholine promotes longevity and fitness in *Caenorhabditis elegans*. Metabolites.

[36] Lozano J., Belles X. (2011). Conserved repressive function of Krüppel homolog 1 on insect metamorphosis in hemimetabolous and holometabolous species. Sci. Rep..

[37] Zhang Q., Dou W., Song Z.H., Jin T.J., Yuan G.R., De Schutter K., Smagghe G., Wang J.J. (2020). Identification and profiling of *Bactrocera dorsalis* microRNAs and their potential roles in regulating the developmental transitions of egg hatching, molting, pupation and adult eclosion. Insect Biochem. Mol. Biol..

[38] Li M., Wang G., Shang R., Xu Q., Zhang J., Sun R., Li L. (2021). Comparative lipid profile analysis of *Hermetia illucens* larvae fed food waste at different days of age using an LC-MS-based lipidomics approach. J. Insect Sci..

[39] Adu-Gyamfi E., Johnson K.A., Fraser M.E., Scott J.L., Soni S.P., Jones K.R., Digman M.A., Gratton E., Tessier C.R., Stahelin R.V. (2015). Host cell plasma membrane phosphatidylserine regulates the assembly and budding of ebola virus. J. Virol..

[40] Suzuki T., Suzuki Y. (2006). Virus infection and lipid rafts. Biol. Pharm. Bull..

[41] Patterson P.H., Acar N., Ferguson A.D., Trimble L.D., Sciubba H.B., Koutsos E.A. (2021). The impact of dietary black soldier fly larvae oil and meal on laying hen performance and egg quality. Poult. Sci..

[42] Navrotskaya V., Wnorowski A., Turski W., Oxenkrug G. (2018). Effect of lynurenic acid on pupae viability of *Drosophila melanogaster* cinnabar and cardinal eye color mutants with altered tryptophan-kynurenine metabolism. Neurotox. Res..

[43] Koch P.B., Behnecke B., Weigmann-Lenz M., Ffrench-Constant R.H. (2000). Insect pigmentation: Activities of beta-alanyldopamine synthase in wing color patterns of wild-type and melanic mutant swallowtail butterfly *Papilio glaucus*. Pigment Cell. Res..

[44] Zornik E., Paisley K., Nichols R. (1999). Neural transmitters and a peptide modulate *Drosophila* heart rate. Peptide.

[45] Kita T., Ozoem F., Azuma M., Ozoe Y. (2013). Differential distribution of glutamate- and GABA-gated chloride channels in the housefly *Musca domestica*. J. Insect Physiol..

[46] Sellin J., Fülle J.B., Thiele C., Bauer R., Bülow M.H. (2020). Free fatty acid determination as a tool for modeling metabolic diseases in *Drosophila*. J. Insect Physiol..

[47] Kaczmarek A., Wrońska A.K., Kazek M., Boguś M.I. (2020). Metamorphosis-related changes in the free fatty acid profiles of *Sarcophaga* (*Liopygia*) *argyrostoma* (Robineau-Desvoidy, 1830). Sci. Rep..

[48] Ren J.L., Dong H., Han Y., Yang L., Zhang A.H., Sun H., Li Y., Yan G., Wang X.J. (2020). Network pharmacology combined with metabolomics approach to investigate the protective role and detoxification mechanism of Yunnan Baiyao formulation. Phytomedicine.

[49] Daniels R.C., Tiba M.H., Cummings B.C., Yap Y.R., Ansari S., McCracken B.M., Sun Y., Jennaro T.S., Ward K.R., Stringer K.A. (2022). Redox potential correlates with changes in metabolite concentrations attributable to pathways active in oxidative stress response in swine traumatic shock. Shock.

[50] Fazio F., Lionetto L., Molinaro G., Bertrand H.O., Acher F., Ngomba R.T., Notartomaso S., Curini M., Rosati O., Scarselli P. (2012). Cinnabarinic acid, an endogenous metabolite of the kynurenine pathway, activates type 4 metabotropic glutamate receptors. Mol. Pharmacol..

[51] Ze L.J., Xu P., Kang W.N., Wu J.J., Jin L., Anjum A.A., Li G.Q. (2021). Disruption of kynurenine pathway reveals physiological importance of tryptophan catabolism in *Henosepilachna vigintioctopunctata*. Amino Acids.

[52] Liu J., Chen Z., Xiao Y., Asano T., Li S., Peng L., Chen E., Zhang J., Li W., Zhang Y. (2021). Lepidopteran wing scales contain abundant cross-linked film-forming histidine-rich cuticular proteins. Commun. Biol..

[53] Mentel M., Ahuja E.G., Mavrodi D.V., Breinbauer R., Thomashow L.S., Blankenfeldt W. (2009). Of two make one: The biosynthesis of phenazines. ChemBioChem.

[54] Matsuoka Y., Monteiro A. (2018). Melanin pathway fenes regulate color and morphology of butterfly wing scales. Cell Rep..

[55] Tomcala A., Tollarová M., Overgaard J., Simek P., Kostál V. (2006). Seasonal acquisition of chill tolerance and restructuring of membrane glycerophospholipids in an overwintering insect: Triggering by low temperature, desiccation and diapause progression. J. Exp. Biol..

[56] Wang J., Ran L.L., Li Y., Liu Y.H. (2020). Comparative proteomics provides insights into diapause program of *Bactrocera minax* (Diptera: Tephritidae). PLoS ONE.

[57] Didion E.M., Sabree Z.L., Kenyon L., Nine G., Hagan R.W., Osman S., Benoit J.B. (2021). Microbiome reduction prevents lipid accumulation during early diapause in the northern house mosquito, *Culex pipiens pipiens*. J. Insect Physiol..

[58] Khanmohamadi F., Khajehali J., Izadi H. (2016). Diapause and cold hardiness of the almond wasp, *Eurytoma amygdali* (Hymenoptera: Eurytomidae), two independent phenomena. J. Econ. Entomol..

[59] Santos J.L., Ebert D. (2022). Trehalose provisioning in *Daphnia* resting stages reflects local adaptation to the harshness of diapause conditions. Biol. Lett..

[60] Huang Q., Zhang G., Nan J., Cheng W., Zhu-Salzman K. (2021). Characterization of trehalose metabolic genes and corresponding enzymatic activities during diapause of *Sitodiplosis mosellana*. J. Insect Physiol..

[61] Song Y., Huang W.W., Zhou Y., Li Z.W., Ji R., Ye X.F. (2021). Physiological characteristics and cold tolerance of overwintering eggs in *Gomphocerus sibiricus* L. (Orthoptera: Acrididae). Arch. Insect Biochem. Physiol..

[62] Tanwar A.K., Kirti J.S., Kumar S., Dhillon M.K. (2021). The amino acid and lipophilic profiles of *Chilo partellus* (Swinhoe) larvae fluctuate with diapause. J. Exp. Zool. A Ecol. Integr. Physiol..

[63] Koštál V., Renault D., Rozsypal J. (2011). Seasonal changes of free amino acids and thermal hysteresis in overwintering heteropteran insect, *Pyrrhocoris apterus*. Comp. Biochem. Physiol. A Mol. Integr. Physiol..

[64] Van der Krieken S.E., van-der Pijl P.C., Lin Y., Popeijus H.E., Mensink R.P., Plat J. (2019). Search for natural compounds that increase apolipoprotein A-I transcription in HepG2 cells: Specific attention for BRD4 inhibitors. Lipids.

[65] Li J., Cao F., Yin H.L., Huang Z.J., Lin Z.T., Mao N., Sun B., Wang G. (2020). Ferroptosis: Past, present and future. Cell Death Dis..

[66] Manaboon M., Yasanga T., Sakurai S., Singtripop T. (2012). Programmed cell death of larval tissues induced by juvenile hormone in the bamboo borer, *Omphisa fuscidentalis*. J. Insect Physiol..

[67] Galles C., Prez G.M., Penkov S., Boland S., Porta E., Altabe S.G., Labadie G.R., Schmidt U., Knölker H.J., Kurzchalia T.V. (2018). Endocannabinoids in *Caenorhabditis elegans* are essential for the mobilization of cholesterol from internal reserves. Sci. Rep..

