# Supplementary material for: Metabolite Changes in Orange Dead Leaf Butterfly Kallima inachus during Ontogeny and Diapause
Source: Metabolites. 2022 Aug 27;12(9):804. doi: 10.3390/metabo12090804 (PMC9501346; doi:10.3390/metabo12090804)
Supplement: Supplementary file 1 [file metabolites-12-00804-s001.zip › Figure S1.pdf]

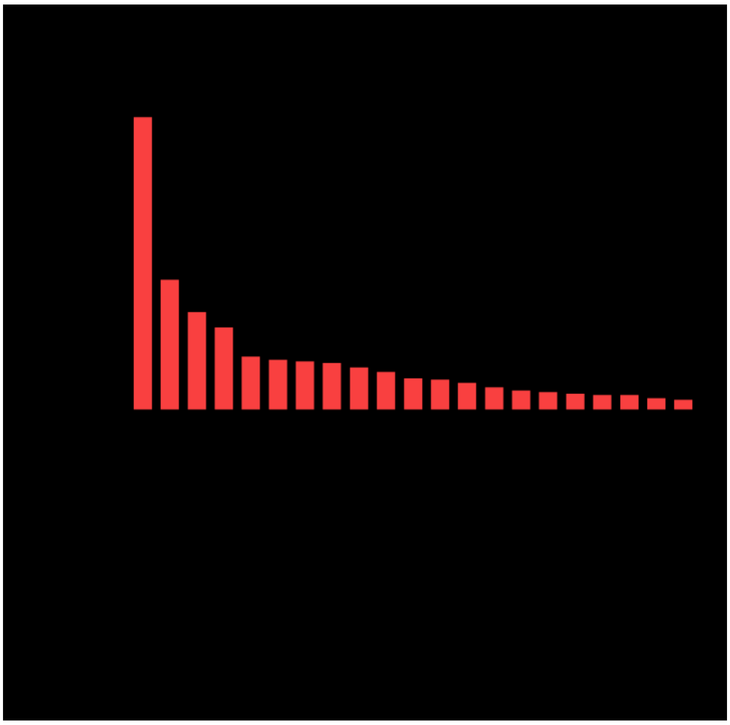

Figure S1. The top 20 largest metabolic secondary categories of all identified metabolites. The Y-axis represents the number of metabolites.
